# Optimal Control Algorithm for Stochastic Systems with Parameter Drift

**DOI:** 10.3390/s23125743

**Published:** 2023-06-20

**Authors:** Xiaoyan Zhang, Song Gao, Chaobo Chen, Jiaoru Huang

**Affiliations:** School of Electronic Information Engineering, Xi’an Technological University, Xi’an 710021, China; xyzhang@xatu.edu.cn (X.Z.); gaos@xatu.edu.cn (S.G.); huangjiaoru@xatu.edu.cn (J.H.)

**Keywords:** stochastic systems, mixed uncertainties, parameter drift, Kalman filter, dual control

## Abstract

A novel optimal control problem is considered for multiple input multiple output (MIMO) stochastic systems with mixed parameter drift, external disturbance and observation noise. The proposed controller can not only track and identify the drift parameters in finite time but, furthermore, drive the system to move towards the desired trajectory. However, there is a conflict between control and estimation, which makes the analytic solution unattainable in most situations. A dual control algorithm based on weight factor and innovation is, therefore, proposed. First, the innovation is added to the control goal by the appropriate weight and the Kalman filter is introduced to estimate and track the transformed drift parameters. The weight factor is used to adjust the degree of drift parameter estimation in order to achieve a balance between control and estimation. Then, the optimal control is derived by solving the modified optimization problem. In this strategy, the analytic solution of the control law can be obtained. The control law obtained in this paper is optimal because the estimation of drift parameters is integrated into the objective function rather than the suboptimal control law, which includes two parts of control and estimation in other studies. The proposed algorithm can achieve the best compromise between optimization and estatimation. Finally, the effectiveness of the algorithm is verified by numerical experiments in two different cases.

## 1. Introduction

Parameter drift refers to the change in parameters in the system when the component exceeds its working life or failure. The occurrence of drift is closely related to the working principle, operating environment and the physical properties of components’ materials [1], especially in aerospace systems. For example, the gyroscope installed on a satellite is the most important measurement tool for measuring the angular velocity of the satellite attitude. However, due to the strong radiation and strong lightning of the space environment, as well as the mechanical torsion, degradation and wear caused by the vibration, material creep and time stress, the structural parameters of a gyroscope will drift over time [2,3]. The acceleration of drift will seriously affect the navigation accuracy of the inertial platform, which may have serious consequences. If the drift parameter of a gyroscope can be accurately known at any time, the drift can be found the fastest so that corresponding measures can be taken to reduce the occurrence of accidents. Therefore, it is necessary to study the system control problem involving parameter drift.

Generally, researchers take parameter drift as an irresolvable uncertainty. There has been some research on this kind of uncertainty. Wen et al. [4] proposed an adaptive control strategy for model predictive control for uncertainties in control systems, including modeling dynamic and bounded uncertainties. Aiming at parameter uncertainty and model uncertainty, a sampling-based approximation method is proposed in [5], which consists of two parts, the dual part of the scenario tree and the exploitation part of computing the open loop control sequence, both of which perform their respective functions and finally obtain a dual predictive control formula. In addition to these model structural uncertainties, there are various uncertainties caused by noise. Most current studies only consider Gaussian white noise; few researchers have studied non-Gaussian noise. Ma et al. [6] consider the case of following thick tail distribution instead of Gaussian distribution, and Ma et al. [7] consider the filtering problem of nonlinear stochastic systems with measured outliers. However, they all have their limitations. Ma L. et al. [7] only studies the filtering problem involving the measured outliers, while Ma X. et al. [6,8] consider both the filtering and control, but the research object is limited to the single input–single output (SISO) system. Up to now, there have been many research results on the control problems of stochastic linear SISO time-invariant systems involving parameter drift. For fast arbitrary drift phenomena, the drift parameters are viewed as unknown augmented states that are compensated via model error compensation in the literature [9]. For the deterministic drift phenomena, a double exponentially weighted moving average feedback controller is designed to compensate the slow drift of the system in the literature [10]. In addition, Yang et al. [11] proposed a suboptimal dual control method for the systems with parameter drifting. However, these above methods are only for SISO cases. For the MIMO system, the common methods are to decouple into multiple SISO systems. However, these methods often overlook a large amount of information in the actual system and cannot accurately describe the actual system [12]. Actually, with the rapid development of science and technology, the structures of modern mechanical systems and aerospace systems have become increasingly complex, with more and more subsystems and functions. Each component is interconnected and the subsystems are coupled with each other, which makes a simple linear SISO model far from enough to meet the current needs. Therefore, it is urgent to solve the control and estimation problem for MIMO systems with parameter drift.

Presently, numerous works have contributed to the research regarding the MIMO system, such as the approximation of nonlinear MIMO system control [13], the optimal adaptive tracking control [14] and stable adaptive control of nonlinear multivariable systems [15], model predictive control [16], adaptive certainty equivalence control [17,18] and so on. For stochastic systems with parameters and noise unicertainties, the idea of dual control was first proposed by former Soviet researcher Feldbaum as early as the early 1960s. He and NASA‘s Barshalom (founder of information fusion) noticed this kind of problem and pointed out in their series of papers that, except for a few ideal cases, the optimal control of such systems generally pursues two conflicting goals: on the one hand, the controller needs to optimize the system. In order to achieve good control effects, the control amount should be small and not too large, which is the cautious role of the controller. On the other hand, the controller also needs to learn to deal with uncertainties regarding parameters and system states. In order to obtain better processing results, it is hoped that the control amount will be as large as possible to motivate rich information in the system [19,20], which is the detection function of the controller. The controller design theory that combines caution and detection is a challenge to the existing theory of LQG, which is the so-called dual adaptive control problem of systems with double uncertainty. These two actions conflict with each other and cannot be carried out separately, resulting in difficulty in obtaining optimal control solutions in most cases. There have been some research results about the problem, such as adaptive dual control [21,22] and robust learning control [23], innovation dual control [24], variance minimization cumulant control under complete statistical characterization [25], LQG nominal dual control [26,27] and so on. The above approaches are designed on the basis of the state space model for the stochastic system with unknown parameters, not emphasizing the correlation and coupling between internal variables and outside variables in the system. In [22], a suboptimal dual control strategy is proposed for stochastic systems with parameter uncertainties. However, the designed controller contains two parts: one is for control and the other is for learning. Among them, the learning part is to add the trace of covariance matrix to the control law in a specific way, resulting in a suboptimal control strategy. Therefore, they cannot be widely applied. Because the separation principle between the filter and the controller is not fulfilled and the controller and observer are mutually coupled in the MIMO stochastic system, it is a significant challenge to obtain the analytic solution of the controller.

Motivated by the above description, for the MIMO system with parameter drift, we design a novel optimal dual control law by viewing drift parameters as parameters that vary with time in finite time instead of drifting off to infinity. The control designed can track the drift parameters on one hand, and it can also drive the system to run towards the desired target. The innovations of this paper can be summarized as follows. 1. This paper designs an optimal control law for drift parameters, which replaces many suboptimal control laws, including control and learning parts, while reducing computational complexity. 2. Due to the drift parameters in the system, the separation principle does not work. In this paper, multi-step optimization is transformed into one-step optimization, and the optimal goal at each moment is achieved online while estimating, ultimately achieving optimal control. 3. Almost all control systems are MIMO systems, and the model in this paper is closer to the actual system. At the same time, it can also solve the optimal control problem of linearized systems with this type of structure, which has a very broad practical application prospect.

The remaining part of the paper is organized as follows. In Section 2, the problem to be solved is presented. In Section 3, we transform the system model and the drift parameters and then estimate and track them using Kalman filter. A novel dual control law with learning property is designed for the control problem in Section 4. In Section 5, two numerical simulation examples are used to verify the effectiveness of the algorithm proposed in this paper, and the conclusion is presented in Section 6.

## 2. Problem Statement

Consider a general multi-input multi-output stochastic system model:

(1) $$\begin{array}{c} \left\{\begin{array}{cc} z_{1}\left(t+1\right)= & g_{11}\left(t\right)z_{1}\left(t\right)+g_{12}\left(t\right)z_{2}\left(t\right)+\cdots +g_{1m}\left(t\right)z_{m}\left(t\right)\\ & +h_{11}\left(t\right)u_{1}\left(t\right)+h_{12}\left(t\right)u_{2}\left(t\right)+\cdots +h_{1r}\left(t\right)u_{r}\left(t\right)+\varepsilon_{1}\left(t\right)\\ z_{2}\left(t+1\right)= & g_{21}\left(t\right)z_{1}\left(t\right)+g_{22}\left(t\right)z_{2}\left(t\right)+\cdots +g_{2m}\left(t\right)z_{m}\left(t\right)\\ & +h_{21}\left(t\right)u_{1}\left(t\right)+h_{22}\left(t\right)u_{2}\left(t\right)+\cdots +h_{2r}\left(t\right)u_{r}\left(t\right)+\varepsilon_{2}\left(t\right)\\ & \vdots \\ z_{m}\left(t+1\right)= & g_{m1}\left(t\right)z_{1}\left(t\right)+g_{m2}\left(t\right)z_{2}\left(t\right)+\cdots +g_{mm}\left(t\right)z_{m}\left(t\right)\\ & +h_{m1}\left(t\right)u_{1}\left(t\right)+h_{m2}\left(t\right)u_{2}\left(t\right)+\cdots +h_{mr}\left(t\right)u_{r}\left(t\right)+\varepsilon_{m}\left(t\right) \end{array}\right. \end{array}$$

where $u_{j}\left(t\right),j=1,2,\cdots ,r$ are *r*-dimension control input, $z_{i}\left(t\right),i=1,2,\cdots ,m$ are *m*-dimension control output, $g_{1i}\left(t\right),\cdots ,g_{mi}\left(t\right),h_{1j}\left(t\right),\cdots ,h_{mj}\left(t\right),i=1,2,\cdots ,m$, j=1,2,⋯,r represent the drift parameters reflecting the physical characteristics in the actual system, $\varepsilon_{1}\left(t\right),\varepsilon_{2}\left(t\right),\cdots ,\varepsilon_{m}\left(t\right)$ are stochastic perturbations acting on the system. In general, they are viewed as mutually independent Gaussian white noises with normal distribution, and expressed as $\varepsilon_{i}∼N\left(0,\sigma_{i}^{2}\right),i=1,2,\cdots ,m$.

For the sake of designing controller, the system (1) can be described by the following *r*-dimension input and *m*-dimension output stochastic system model:

(2) $$\begin{array}{c} z(t+1)=G(t)z(t)+H(t)u(t)+\varepsilon (t) \end{array}$$

where z(t) is an output vector composed of each output component $z_{i}\left(t\right),i=1,2,\cdots ,m$, expressed as z(t)=${\left[z_{1}\left(t\right),z_{2}\left(t\right),\cdots ,z_{m}\left(t\right)\right]}^{T}$, *T* represents the transpose of the vector. u(t) is the same denoted by $u\left(t\right)=[u_{1}\left(t\right),u_{2}\left(t\right)$$\cdots ,u_{r}{\left(t\right)]}^{T}$, stochastic perturbations are ε(t) denoted by ε(t)=$[\varepsilon_{1}\left(t\right),$$\varepsilon_{2}\left(t\right),\cdots ,\varepsilon_{m}{\left(t\right)]}^{T}$. Since each component is subject to Gaussian white noise, they satisfy $\varepsilon_{i}∼N\left(0,\Sigma_{\varepsilon }\right)$. Further, $\Sigma_{\varepsilon }=diag\left(\sigma_{1}^{2},\sigma_{2}^{2},\cdots ,\sigma_{m}^{2}\right)$, diag(·) represents the diagonal matrix. For the convenience of writing, *a* is used instead of a(t) in this paper to represent the time-varying parameter with drift. Therefore, the parameters of the system can be rewritten as

$$G=\left[\begin{array}{cccc} g_{11} & g_{12} & \cdots & g_{1m}\\ g_{21} & g_{22} & \cdots & g_{2m}\\ \vdots & \vdots & \cdots & \vdots \\ g_{m1} & g_{m2} & \cdots & g_{mm} \end{array}\right]\;H=\left[\begin{array}{cccc} h_{11} & h_{12} & \cdots & h_{1r}\\ h_{21} & h_{22} & \cdots & h_{2r}\\ \vdots & \vdots & \cdots & \vdots \\ h_{m1} & h_{m2} & \cdots & h_{mr} \end{array}\right]$$

It is worth noting that rewritten model (2) and original model (1) are interchangeable and have the same structure.

Next, the performance index is expressed in linear quadratic form as follows:

(3) $$J≜E\left\{\sum_{t}^{N-1}{\left[z\left(t+1\right)-z_{r}\left(t+1\right)\right]}^{T}Q\left[z\left(t+1\right)-z_{r}\left(t+1\right)\right]\right\}$$

where $z_{r}\left(t+1\right),t=0,1,\cdots ,N-1$ is the desired output trajectory, $Q=diag[q_{1},q_{2},\cdots ,q_{m}]$ is a semidefinite diagonal weight matrix. E{·} represents the mathematical expectation of error between the actual output and the desired output based on under the information set in the past.

For the above model (2), the goal of our research is to look for an optimal control to minimize the output deviation of the system in statistical sense. At the same time, it can effectively deal with the drift parameters in the system. The control problem to be solved in this paper can be described as the following optimization problem:

$$\begin{array}{cc} (P)\; & \underset{u}{min}J\\ \; & s.t.z(t+1)=G(t)z(t)+H(t)u(t)+\varepsilon (t)\\ \; & t=0,1,2,\cdots ,N-1 \end{array}$$

For the above optimization problem (*P*), when the parameters representing the characteristics of the system are all known, we can treat it as given constant and no parameter drift at any stage; the traditional minimum variance approach has been solved, and it is quite mature. When parameters are drifting, it is a new problem and the existing approach cannot be solved. The work of this paper is to derive a control law for the above dual control problem so that the derived control law can predict or track effectively the drift parameters while driving the system to work towards the desired target.

## 3. Parameter Prediction

In the problem (*P*), parameter drift makes it difficult to determine the control law. Therefore, the problem of parameter estimation is given priority to solve. The model (2) is transformed into the following model:

(4) z(t+1)=Φ(t)Θ(t)+ε(t)

where z(t+1) is the output vector at time t+1, Φ(t)=diag[ϕ(t),ϕ(t),⋯,ϕ(t)]; here, ϕ(t) as a new system vector containing the output component and the control component, denoted by $\phi \left(t\right)=[z^{T}\left(t\right),u^{T}\left(t\right)]$, Θ(t), is the parameter vector consisting of all the drift parameters, denoted by $\Theta \left(t\right)=[\theta_{1}^{T}\left(t\right),\theta_{2}^{T}\left(t\right),$$\cdots ,\theta_{m}^{T}{\left(t\right),]}^{T}$, $\theta_{i}\left(t\right)={\left[g_{i1},g_{i2},\cdots ,g_{im},h_{i1},h_{i2},\cdots ,h_{ir}\right]}^{T}$, i=1,2,⋯,m.

In the above model (4), the parameter vector Θ is assumed to satisfy Gaussian distribution with initial mean $\hat{\Theta }\left(0\right)$ and initial covariance matrix P(0). The disturbance noise is assumed to be mutually independent with the parameter vector.

In fact, no matter in the actual aerospace system, high-speed train operation system or large building structure, bridge or ship system, there are some components of failure, wear and aging phenomena, which are reflected in the change in system parameters. At the same time, there are also some systems whose parameters are ideal values obtained from trials, but, due to the complexity of the working environment, the one-to-one correspondence between the real parameters and the ideal values is not established, or they inevitably drift due to production errors, external time stresses and physical properties of materials. The drift is a dynamic process and affected by external noise or environment. A dynamic model of drift parameters is therefore established:

(5) Θ(t+1)=Γ(t)Θ(t)+ξ(t)

where Γ represents the drift coefficient matrix that can reflect the amplitude or changing trend in the parameter drift. ξ(t) represents the process noise during the operation of the system. To use Kalman filtering, we assume that it is independent of the measurement noise and follows normal distribution, namely ε(t) satisfies $\xi ∼N(0,\Sigma_{\xi })$.

When the drift coefficient is unknown but constant, it is shown from (5) that the drift parameters are stable during the operation of the system, which is an ideal condition. At the moment, Γ is the identity matrix. When the parameters are fluctuant, Γ is not identity matrix. If ${\left||\Gamma |\right|}_{2}$ is lager than 1, it indicates that the value of the system parameters gradually increases over time. For example, in the motor speed control system, with the continuous operation of the motor, the motor temperature is more and more high as time goes by and the resistance becomes great as the temperature increases. If ${\left||\Gamma |\right|}_{2}$ is smaller than 1, it indicates that the value of the system parameters gradually decreases over time; for example, as the key measuring device of aerospace attitude control system, the performance of gyroscope is degraded due to wear and creep caused by harsh external environment and time stress. When the gyroscope parameter value of drift is less than the preset threshold value, the drift coefficient can be adjusted appropriately by modifying its torque to eliminate errors as much as possible. However, because the wear and aging caused by time stress is irreversible, the modified drift parameters still show a decreasing trend; namely, the drift parameter decreases gradually. The above analysis shows the situation that the parameters are time-varying, and the tendency of parametric variation in the model (5) is described at the same time. Therefore, above model (5) has universality.

Since the past control and output information is needed in processing drift parameters and solving optimal control, all information collected by the control law before the sampling time *t* is called real-time information set, that is:

$$\begin{array}{cc} z^{t}= & \{z_{1}\left(0\right),\cdots ,z_{m}\left(0\right),u_{1}\left(0\right),\cdots ,u_{r}\left(0\right),z_{1}\left(1\right),\cdots ,z_{m}\left(1\right),u_{1}\left(1\right),\cdots ,u_{r}\left(1\right),\cdots ,\\ \; & z_{1}\left(t-1\right),\cdots ,z_{m}\left(t-1\right),u_{1}\left(t-1\right),\cdots ,u_{r}\left(t-1\right),z_{1}\left(t\right),\cdots ,z_{m}\left(t\right)\}. \end{array}$$

If the initial time of the control system starts from the time t=1, the initial information set is represented as $z^{1}=\left\{z_{1}\left(1\right),z_{2}\left(1\right),\cdots ,z_{m}\left(1\right),u_{1}\left(0\right),u_{2}\left(0\right),\cdots ,u_{r}\left(0\right)\right\}$, which is set up in advance before the system runs.

In the practical system, $z^{t}$ is known, the parameter estimation, estimate error and estimation covariance matrix are defined:

(6) $$\begin{array}{ccc} \hat{\Theta }\left(t|t\right) & = & E\{\Theta \left(t\right)|z^{t}\} \end{array}$$

(7) $$\begin{array}{ccc} \tilde{\Theta }\left(t|t\right) & = & \Theta \left(t\right)-\hat{\Theta }\left(t|t\right) \end{array}$$

(8) $$\begin{array}{ccc} P(t|t) & = & E\{\tilde{\Theta }\left(t|t\right)\tilde{\Theta }{\left(t|t\right)}^{T}\} \end{array}$$

where E{·} represents mathematical expectation.

In the system described by (4) and (5), the evolutions of the conditional mean and covariance matrix are given by the standard Kalman filter equations

(9) $$\begin{array}{cc} \hat{\Theta }\left(t+1|t\right) & =\Gamma \hat{\Theta }\left(t|t\right) \end{array}$$

(10) $$\begin{array}{cc} \hat{\Theta }\left(t+1|t+1\right) & =\hat{\Theta }\left(t+1|t\right)+F\left(t+1\right)e\left(t+1\right) \end{array}$$

(11) $$\begin{array}{cc} e(t+1) & =z(t+1)-\Phi \left(t\right)\hat{\Theta }\left(t+1|t\right) \end{array}$$

(12) $$\begin{array}{cc} F(t+1) & =\frac{P\left(t+1|t\right)\Phi^{T}\left(t\right)}{\left[\Phi \left(t\right)P\left(t+1|t\right)\Phi^{T}\left(t\right)+\Sigma_{\xi }\right]} \end{array}$$

(13) $$\begin{array}{cc} P(t+1|t) & =\Gamma P\left(t|t\right)\Gamma^{T}+\Sigma_{\varepsilon } \end{array}$$

(14) $$\begin{array}{cc} P(t+1|t+1) & =P(t+1|t)-F(t+1)\Phi (t)P(t+1|t) \end{array}$$

where (11) is the new information about the system parameters contained in z(t+1), i.e., the innovation sequence.

Equation (10) shows the estimation and tracking of drift parameters at the current sanpling isntant.

## 4. Main Results

In general, it is easy to obtain the optimal control sequence $u^{*}\left(t\right)$ by minimizing the performance index at each stage using dynamic programming. However, the realization condition of dynamic programming is that the system parameters must be known; otherwise, the dynamic programming cannot be recursive. In this paper, it is easy to find that it is very intractable to obtain the optimal control law of the system by direct dynamic programming because of system parameter drift and uncertainties. Therefore, we design a novel MIMO dual control optimization algorithm. The control law designed not only can track the drift parameters of the system but can also drive the control system to run towards the desired target, i.e., a tradeoff between the control objectives and the parameter estimation objectives.

Different from [22], the part dealing with drift parameters is directly integrated into the performance index with different weight factors. In this way, the control law obtained can be guaranteed to be the optimal control law, but it also greatly reduces the amount of calculation, which is very convenient for application and popularization.

In order to realize the above two objectives, we need to simplify the initial problem. The principle of simplification is that the controller solved has dual characteristics and can obtain the analytic solution. In order to obtain the analytic solution, the general solution is to convert the global optimal to the single-step optimal so that not only the parameter drift can be taken into account but also the optimal control of the system can be obtained through a series of processing methods. Therefore, we transform the overall performance index to the single-step optimal performance index; the new performance index is rewritten as:

(15) $$\begin{array}{c} \begin{array}{cc} J_{t}= & E\{{\left[z\left(t+1\right)-z_{r}\left(t+1\right)\right]}^{T}Q\left[z\left(t+1\right)-z_{r}\left(t+1\right)\right]\\ & -\beta e^{T}\left(t+1\right)Qe\left(t+1\right)\left|z^{t}\right\},\\ & t=0,1,2,\cdots ,N-1. \end{array} \end{array}$$

where β is the learning weighting factor.

The first term of Equation (15) considers the control ability of the controller and guarantees the system to track the reference signal in the optimal way. The second term endows the learning ability of the controller and carries out prediction output of the model to inch as close as possible to the practical output of the system. The sign of the second is thus negative because of the mutual conflict between optimization and estimation. It can be seen that the control law determined by the performance index (15) has dual characteristics.

The optimal weight coefficient β determined indicates that the control law derived by the performance index (15) can achieve the best tradeoff between optimization and estimation. In addition, we can obtain the analytic solution of the control law using (15), and the specific derivation process is as follows:

(16) $$\begin{array}{c} \begin{array}{cc} & E\{e^{T}\left(t+1\right)Qe\left(t+1\right)\}\\ = & E\{{\left[z\left(t+1\right)-\Phi \left(t\right)\hat{\Theta }\left(t\right)\right]}^{T}Q\left[z\left(t+1\right)-\Phi \left(t\right)\hat{\Theta }\left(t\right)\right]\}\\ = & E\{{\left[\Phi \left(t\right)\Theta \left(t\right)+\varepsilon \left(t\right)-\Phi \left(t\right)\hat{\Theta }\left(t\right)\right]}^{T}Q\left[\Phi \left(t\right)\Theta \left(t\right)+\varepsilon \left(t\right)-\Phi \left(t\right)\hat{\Theta }\left(t\right)\right]\}\\ = & E\{{\left[\Phi \left(t\right)\tilde{\Theta }\left(t\right)+\varepsilon \left(t\right)\right]}^{T}Q\left[\Phi \left(t\right)\tilde{\Theta }\left(t\right)+\varepsilon \left(t\right)\right]\}\\ = & E\left\{{\tilde{\Theta }}^{T}\left(t\right)\Phi^{T}\left(t\right)Q\Phi \left(t\right)\tilde{\Theta }\left(t\right)\right\}+tr\left(Q\Sigma_{\varepsilon }\right)\\ = & tr\left[\Phi^{T}\left(t\right)Q\Phi \left(t\right)P\left(t\right)\right]+tr\left(Q\Sigma_{\varepsilon }\right) \end{array} \end{array}$$

where tr(·) represents the trace of the matrix. The theorem tr(ABC)=tr(BCA)=tr(CAB) is used in Equation (16).

The following equation can be obtained by Equations (15) and (16):

(17) $$\begin{array}{c} \begin{array}{cc} J_{t} & ={\left[\Phi \left(t\right)\hat{\Theta }\left(t\right)-z_{r}\left(t+1\right)\right]}^{T}Q\left[\Phi \left(t\right)\hat{\Theta }\left(t\right)-z_{r}\left(t+1\right)\right]\\ & +\left(1-\beta \right)[tr\left(\Phi^{T}\left(t\right)Q\Phi \left(t\right)P\left(t\right)\right)+tr\left(Q\Sigma_{\varepsilon }\right)] \end{array} \end{array}$$

To deal with the optimal control problem, introduce the following partitionings of the observation vector Φ(t), the estimated drift parameter vector ${\hat{\theta }}_{i}\left(t\right)$ and covariance matrix P(t):

(18) $$\begin{array}{c} {\hat{\theta }}_{i}^{T}\left(t\right)=\left[{\hat{g}}_{i}^{T},{\hat{h}}_{i}^{T}\right],i=1,2,\cdots ,m \end{array}$$

(19) $$P\left(t\right)=\left[\begin{array}{cccc} P_{11}\left(t\right) & P_{12}\left(t\right) & \cdots & P_{1r}\left(t\right)\\ P_{21}\left(t\right) & P_{22}\left(t\right) & \cdots & P_{2r}\left(t\right)\\ \vdots & \vdots & \cdots & \vdots \\ P_{m1}\left(t\right) & P_{m2}\left(t\right) & \cdots & P_{mm}\left(t\right) \end{array}\right]$$

where ${\hat{g}}_{i}={\left[{\hat{g}}_{i1},{\hat{g}}_{i2},\cdots ,{\hat{g}}_{im}\right]}^{T}$, ${\hat{h}}_{i}={\left[{\hat{h}}_{i1},{\hat{h}}_{i2},\cdots ,{\hat{h}}_{ir}\right]}^{T}$, $P_{11}\left(t\right),P_{22}\left(t\right),\cdots ,P_{mm}\left(t\right)$ are (m+r)−dimension square matrices. $P_{ii},i=1,2,\cdots ,m$ is, therefore, partitioned and written as

(20) $$P_{ii}\left(t\right)=\left[\begin{array}{cc} P_{gi}\left(t\right) & P_{ghi}\left(t\right)\\ P_{ghi}^{T}\left(t\right) & P_{hi}\left(t\right) \end{array}\right],i=1,2,\cdots ,m$$

where $P_{gi}\left(t\right)$ is the m−dimension square matrix, $P_{hi}\left(t\right)$ is the r−dimension square matrix.

Combining Equations (18)–(20):

(21) $$\begin{array}{c} \begin{array}{cc} & tr[\Phi^{T}\left(t\right)Q\Phi \left(t\right)P\left(t\right)]\\ = & tr[diag\left(\phi^{T}\left(t\right),\phi^{T}\left(t\right),\cdots ,\phi^{T}\left(t\right)\right)Qdiag\left(\phi \left(t\right),\phi \left(t\right),\cdots ,\phi \left(t\right)\right)P\left(t\right)]\\ = & tr[\Phi^{T}\left(t\right)q_{1}\phi \left(t\right)P_{11}\left(t\right)+\Phi^{T}\left(t\right)q_{2}\phi \left(t\right)P_{22}\left(t\right)+\cdots +\Phi^{T}\left(t\right)q_{m}\phi \left(t\right)P_{mm}\left(t\right)]\\ = & \sum_{i=1}^{m}tr\left[\phi^{T}\left(t\right)q_{i}\phi \left(t\right)P_{ii}\left(t\right)\right]\\ = & \sum_{i=1}^{m}tr\left[z\left(t\right)q_{i}z^{T}\left(t\right)P_{gi}\left(t\right)+z\left(t\right)q_{i}u^{T}\left(t\right)P_{ghi}^{T}\left(t\right)+u\left(t\right)q_{i}z^{T}\left(t\right)P_{ghi}\left(t\right)+u\left(t\right)q_{i}u^{T}\left(t\right)P_{hi}\left(t\right)\right] \end{array} \end{array}$$

Substituting Equations (18) and (19) into the first of the performance index (15):

(22) $$\begin{array}{c} \begin{array}{cc} & {\left[\Phi \left(t\right)\hat{\Theta }\left(t\right)-z_{r}\left(t\right)\right]}^{T}Q\left[\Phi \left(t\right)\hat{\Theta }\left(t\right)-z_{r}\left(t\right)\right]\\ = & \sum_{i=1}^{m}\left[{\hat{g}}_{i}^{T}z\left(t\right)q_{i}z^{T}\left(t\right){\hat{g}}_{i}+{\hat{g}}_{i}^{T}z\left(t\right)q_{i}u^{T}\left(t\right){\hat{h}}_{i}+{\hat{h}}_{i}^{T}u\left(t\right)q_{i}z^{T}\left(t\right){\hat{a}}_{i}+{\hat{h}}_{i}^{T}u\left(t\right)q_{i}u^{T}\left(t\right){\hat{h}}_{i}\right]\\ & -\sum_{i=1}^{m}\left[y_{ri}^{T}\left(t+1\right)q_{i}\left(z^{T}\left(t\right){\hat{a}}_{i}+u^{T}\left(t\right){\hat{h}}_{i}\right)\right]\\ & -\sum_{i=1}^{m}\left[\left({\hat{g}}_{i}^{T}z\left(t\right)+{\hat{h}}_{i}^{T}u\left(t\right)\right)q_{i}z_{ri}\left(t+1\right)\right]+z_{r}^{T}\left(t+1\right)Qz_{r}\left(t+1\right) \end{array} \end{array}$$

Combining Equations (21) and (22), the target function is derived as follows:

(23) $$\begin{array}{c} \begin{array}{cc} J_{t} & =\sum_{i=1}^{m}\left[{\hat{g}}_{i}^{T}z\left(t\right)q_{i}z^{T}\left(t\right){\hat{g}}_{i}+{\hat{a}}_{i}^{T}z\left(t\right)q_{i}u^{T}\left(t\right){\hat{h}}_{i}+{\hat{h}}_{i}^{T}u\left(t\right)q_{i}z^{T}\left(t\right){\hat{g}}_{i}+{\hat{h}}_{i}^{T}u\left(t\right)q_{i}u^{T}\left(t\right){\hat{h}}_{i}\right]\\ & -\sum_{i=1}^{m}\left[z_{ri}^{T}\left(t+1\right)q_{i}\left(z^{T}\left(t\right){\hat{g}}_{i}+u^{T}\left(t\right){\hat{h}}_{i}\right)\right]\\ & -\sum_{i=1}^{m}\left[\left({\hat{g}}_{i}^{T}z\left(t\right)+{\hat{h}}_{i}^{T}u\left(t\right)\right)q_{i}z_{ri}\left(t+1\right)\right]+z_{r}^{T}\left(t+1\right)Qz_{r}\left(t+1\right)\\ & +\left(1-\beta \right)\{\sum_{i=1}^{m}tr[z\left(t\right)q_{i}z^{T}\left(t\right)P_{ai}\left(t\right)+z\left(t\right)q_{i}u^{T}\left(t\right)P_{ghi}^{T}\left(t\right)\\ & +u\left(t\right)q_{i}z^{T}\left(t\right)P_{ghi}\left(t\right)+u\left(t\right)q_{i}u^{T}\left(t\right)P_{bi}\left(t\right)]+tr\left(Q\Sigma_{\varepsilon }\right)\} \end{array} \end{array}$$

In order for the controller to minimize the performance index, the control law is obtained by $\frac{\partial J_{t}}{\partial u_{t}}=0$; it yields

(24) $$u^{*}\left(t\right)=-\frac{\sum_{i=1}^{m}\left[{\hat{h}}_{i}q_{i}{\hat{g}}_{i}^{T}z\left(t\right)-{\hat{b}}_{i}q_{i}z_{ri}\left(t+1\right)\right]+\frac{1-\beta }{2}\sum_{i=1}^{m}P_{ghi}^{T}\left(t\right)z\left(t\right)q_{i}}{\sum_{i=1}^{m}{\hat{h}}_{i}q_{i}{\hat{h}}_{i}^{T}+q_{i}\left(1-\beta \right)\sum_{i=1}^{m}P_{hi}\left(t\right)}$$

Equation (24) is the optimal controller $u^{*}\left(t\right)$ solved for the problem (*P*). It is shown from the above Equation (24) that the controller is not only related to the parameter estimated and estimation covariance matrix but also to the value of β. If the system parameters are constant, the estimation covariance matrix is zero; i.e., the second term of the numerator and denominator in (24) is zero. The contribution of the paper is endowing the learning characteristic of the controller by adding the terms about estimation covariance matrix. The value of β can be determined by the following property.

**Theorem** **1** (Property). In Equation (24), i.e., the optimal control $u^{*}\left(t\right)$, there exists a constant Δ such that 0<β<Δ.

**Proof** **of** **Theorem** **1.** In the practical system, the ability of parameter learning is related to the covariance matrix and the innovation of Kalman filter. The mathematical expectation (16) of innovation square can be obtained. Combining Equations (18)–(20), Equation (23) can be derived.

(25) $$\begin{array}{c} \begin{array}{cc} J_{t} & =\sum_{i=1}^{m}{\hat{g}}_{i}^{T}u\left(t\right)q_{i}u^{T}\left(t\right){\hat{g}}_{i}+\left(1-\beta \right)\sum_{i=1}^{m}tr\left(u\left(t\right)q_{i}u^{T}\left(t\right)P_{hi}\left(t\right)\right)\\ & +\sum_{i=1}^{m}\left[{\hat{g}}_{i}^{T}z\left(t\right)q_{i}u^{T}\left(t\right){\hat{h}}_{i}+{\hat{h}}_{i}^{T}u\left(t\right)q_{i}z^{T}\left(t\right){\hat{g}}_{i}\right]-\sum_{i=1}^{m}\left[z_{ri}^{T}\left(t+1\right)q_{i}\left(z^{T}\left(t\right){\hat{g}}_{i}+u^{T}\left(t\right){\hat{h}}_{i}\right)\right]\\ & +\left(1-\beta \right)\sum_{i=1}^{m}tr\left[z\left(t\right)q_{i}u^{T}\left(t\right)P_{ghi}^{T}\left(t\right)+u\left(t\right)q_{i}z^{T}\left(t\right)P_{ghi}\left(t\right)\right]\\ & -\sum_{i=1}^{m}\left[\left({\hat{g}}_{i}^{T}z\left(t\right)+{\hat{h}}_{i}^{T}u\left(t\right)\right)q_{i}z_{ri}\left(t+1\right)\right]\\ & +\sum_{i=1}^{m}{\hat{g}}_{i}^{T}z\left(t\right)q_{i}z^{T}\left(t\right){\hat{g}}_{i}+z_{r}^{T}\left(t+1\right)Qz_{r}\left(t+1\right)\\ & +\left(1-\beta \right)\{\sum_{i=1}^{m}tr\left[z\left(t\right)q_{i}z^{T}\left(t\right)P_{gi}\left(t\right)\right]+tr\left(Q\Sigma_{\varepsilon }\right)\} \end{array} \end{array}$$

Calculate the trace of Equation (25):

(26) $$\begin{array}{c} J_{t}=\left(\sum_{i=1}^{m}q_{i}\sum_{i=1}^{m}{\hat{h}}_{i}^{T}{\hat{h}}_{i}+\left(1-\beta \right)\sum_{i=1}^{m}q_{i}\sum_{i=1}^{m}trP_{hi}\left(t\right)\right)u^{T}\left(t\right)u\left(t\right)+\zeta_{1}+\zeta_{2} \end{array}$$

where $\zeta_{1}$ denotes the single term containing u(t) or $u^{T}\left(t\right)$, $\zeta_{2}$ denotes the constant term not containing u(t) or $u^{T}\left(t\right)$. Obviously, in order to obtain the minimum value of Equation (26) on u(t), the coefficient of quadratic term should be greater than zero:

$$\sum_{i=1}^{m}q_{i}\sum_{i=1}^{m}{\hat{h}}_{i}^{T}{\hat{h}}_{i}+\left(1-\beta \right)\sum_{i=1}^{m}q_{i}\sum_{i=1}^{m}trP_{hi}\left(t\right)\geq 0$$

namely:

(27) $$\beta \leq 1+\frac{\sum_{i=1}^{m}q_{i}\sum_{i=1}^{m}{\hat{b}}_{i}^{T}{\hat{b}}_{i}}{\sum_{i=1}^{m}q_{i}\sum_{i=1}^{m}trP_{hi}\left(t\right)}$$

Suppose $\Delta =1+\frac{\sum_{i=1}^{m}q_{i}\sum_{i=1}^{m}{\hat{h}}_{i}^{T}{\hat{h}}_{i}}{\sum_{i=1}^{m}q_{i}\sum_{i=1}^{m}trP_{hi}\left(t\right)}$.

The controller performs the estimation to the drift parameters at each stage; the N−1 steps are accumulated to control system. Therefore, the upper bound Δ of β for all inequalities can be obtained by taking the maximum $\sum_{i=1}^{m}trP_{hi}$ in Equation (27). □

## 5. Numerical Experiments

The novel MIMO dual control optimazation algorithm can be obtained by summarizing the above methods:

Step 1: Initialization, and set t=0;

Step 2: Estimate the drift parameters Θ using Kalman filtering (7a–7f);

Step 3: Calculate the optimal control $u^{*}\left(t\right)$, minimizing the performance index using Equation (24).

Step 4: Apply the control $u^{*}\left(t\right)$ to the system (4).

Step 5: If t=N−1, stop; otherwise, set t=t+1; go back to Step 1.

Due to the randomness and unknowability of the system drift, in order to better verify and compare the algorithms in this paper, we treat the drift parameters as a time-varying function and process them. In actual industrial production, due to the continuous changes in operating conditions, the system structural parameters may not be consistent with the label values or ideal values. Therefore, we first treat the drift parameters as unknown but constants for estimation. For example, when the actuator is stuck, the system parameters drift to a fixed value. After that, the parameters are unknown and change in a certain trend over time, such as gyroscopes with continuously deteriorating performance. During the entire lifecycle of the gyroscope, when a certain practice is exceeded, the performance of the gyroscope components shows a certain trend of degradation, which can be considered as an unknown time-varying parameter. This trend is currently being studied in the fields of life prediction and health management.

In response to these two cases, we use two numerical experiments to verify the effectiveness of the control law designed. This section mainly presents simulation results and optimal performance for two cases, and analyzes the simulation results. The system equations in all examples fit the MIMO structure of (1), where r=2, m=2.

The performance index:

$$J=E\{{\left[z\left(t+1\right)-z_{r}\left(t+1\right)\right]}^{T}Q\left[z\left(t+1\right)-z_{r}\left(t+1\right)\right]\}$$

where $Q=I_{2}$, $I_{2}$ denotes 2-order identity matrix, $z_{r}\left(t+1\right)$ are 2-dimensional zero vectors.

Since the drift parameters of the practical system with noise are fluctuating at random, the parameters in every moment are varying, which makes it difficult to show the estimation ability of the controller designed in the paper. In addition, how to drift and the form of drift are not within the scope of this paper. This paper only considers the impact of the system on the parameters after it occurs. Next, let us first consider the first case and use the following simulation example to verify the designed control law.

**Example** **1.** *A simple example is given to illustrate the implementation of the MIMO control algorithm proposed in this paper. Considering a 2-dimensional input and 2-dimensional output system (2), the parameter values after drift are set as follows:*

$$\theta_{1}={\left[0.2,1.8,-0.8,0.7\right]}^{T};\;\;\theta_{2}={\left[-0.6,0.5,0.2,1.5\right]}^{T};$$

*In order to make Kalman filter play a better role, it is necessary to find a suitable initial for prediction and update. Therefore, the initial is set to ${\hat{\theta }}_{1}\left(0\right)={\left[0.1,0.1,0.1,0.1\right]}^{T}$, ${\hat{\theta }}_{2}\left(0\right)=[0.1,$${0.1,0.1,0.1]}^{T}$. External disturbances and observation noise are Gaussian white noise with mean value 0 and variance $\Sigma_{\xi }$, $\Sigma_{\varepsilon }$, respectively, specifically $\Sigma_{\xi }=0.02$, $\sigma_{1}^{2}=\sigma_{2}^{2}=0.2$, $\Sigma_{\varepsilon }=diag\left(\sigma_{1}^{2},\sigma_{2}^{2}\right)$. In addition, the initial covariance matrix is set as $P\left(0\right)=I_{8}$, where $I_{8}$ denotes 8-order identity matrix.*

**Figure 1:**
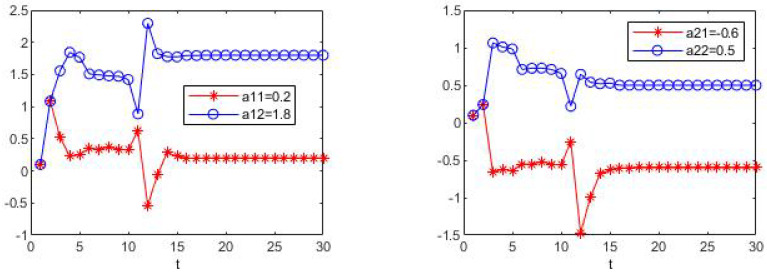
The estimate process of parameters $a_{11}$, $a_{12}$, $a_{21}$ and $a_{22}$.

**Figure 2:**
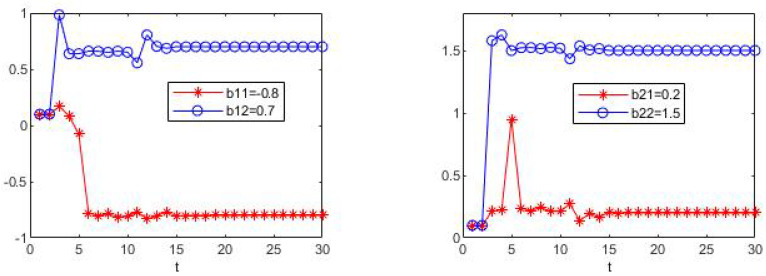
The estimate process of parameters $b_{11}$, $b_{12}$, $b_{21}$ and $b_{22}$.

The simulation results are shown in Figure 1 and Figure 2. In these two figures, the estimation processes of fixed drift parameters are shown. Since the stochastic system is considered in this paper, the estimation process is different every time it is run, but it will eventually reach the true value without exception. At the same time, through multiple runs, we found that, for each simulation, the true values of drift parameters can be accurately estimated before time t=15 and remain stable, which proves the effectiveness of the algorithm in the above case.

The above are the simulation results for the first case. In order to enhance the persuasiveness of our method, we conducted simulations for the second case. Next, we considered that the drift parameters are no longer invariant when unknown but a time-varying function.

**Example** **2.** *Similarly, we consider a dynamic stochastic system with the same structure of (1); the time-varing drift parameters are expressed in the following:*

$$\begin{array}{c} \begin{array}{cc} a_{11} & =20sin\frac{2\pi t}{100},\;a_{12}=50sin\frac{2\pi t}{80}\\ a_{21} & =20sin\frac{2\pi t}{50},\;a_{22}=30sin\frac{2\pi t}{30}\\ b_{11} & =200sin\frac{2\pi t}{100},\;b_{12}=100sin\frac{2\pi t}{80}\\ b_{21} & =100sin\frac{2\pi t}{80},\;b_{22}=200sin\frac{2\pi t}{100} \end{array} \end{array}$$

For convenience, in this example, external disturbances and observation noise still use the variance value from Example 1. At the same time, the initial covariance matrix P(0) remains unchanged.

**Figure 3:**
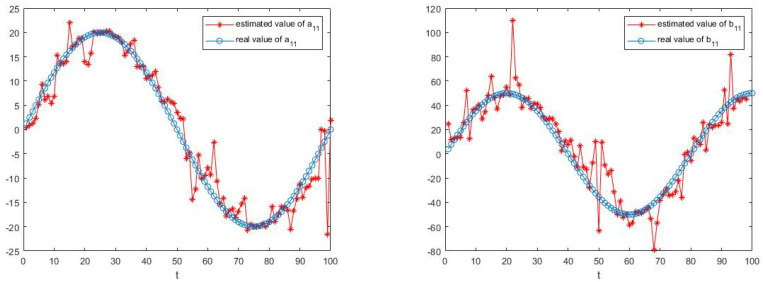
The estimate process of parameters $a_{11}$ and $a_{12}$.

**Figure 4:**
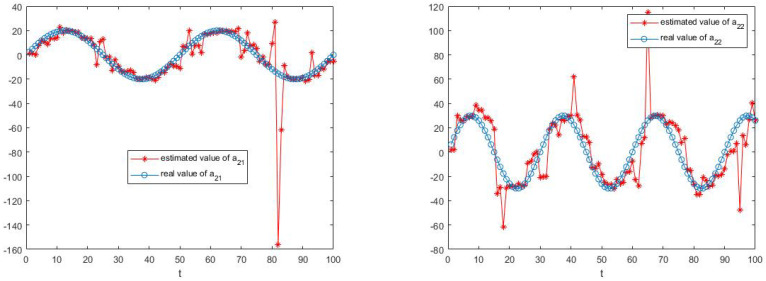
The estimate process of parameters $a_{21}$ and $a_{22}$.

**Figure 5:**
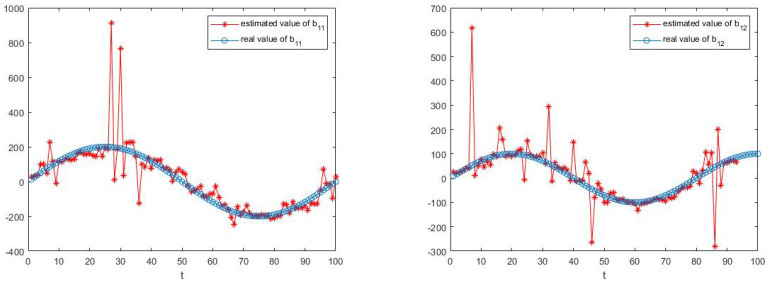
The estimate process of parameters $b_{11}$ and $b_{12}$.

**Figure 6:**
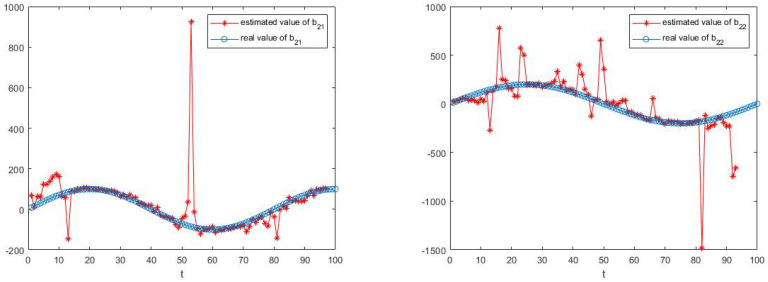
The estimate process of parameters $b_{21}$ and $b_{22}$.

The simulation results are shown in Figure 3, Figure 4, Figure 5 and Figure 6.

Figure 3, Figure 4, Figure 5 and Figure 6 show the learning process of parameters in the last case. It can be seen from the figures that learned parameters can follow true parameters with time, but some errors exist, which is inevitable. This is because learning is a process, which can be shown in Figure 1 and Figure 2, and it needs to take about time 15 to learn the true parameter for each parameter. However, the parameters in this example are changing with time *t* and it is impossible to learn exact values at every moment. However, the algorithm in this paper can estimate approximate parameters and follow the variation trend of parameters. The simulation results prove that the algorithm of this paper provides a feasible method for solving such problems.

In the sequent, the value of performance index under the action of the optimal control law can be calculated by Monte Carlo experiments. Simultaneously, the values under the action of nominal control and pure control are also calculated. The comparison results are shown in Table 1 and Table 2.

As we can see from Table 1 and Table 2, the value of the performance index is different under the action of two different control laws. Among them, the performance index value of pure control is the largest, while that of dual control is the smallest. This indicates that the algorithm proposed is obviously better than the other two, which proves the effectiveness of the designed control law.

In this section, the effectiveness of the proposed optimal dual control strategy is verified by performing two different numerical experiments.

## 6. Conclusions

For the stochastic system involved in parameter drift, disturbances and measurement noise, a novel MIMO dual control strategy is designed in this paper. The proposed control algorithm can not only estimate the drift parameters but can also carry out the optimal control of the system. Due to the conflict between parameter prediction and control target, a weighting factor is added in the preceding parameter term to balance above conflict. The real-time information in the optimal controller designed in the paper is a set of output information and control information at the previous moment. Because of the mutual couple and complex nonliear structure of the MIMO system, the dual control problem based on deep learning is our main research problem in the future.

## Figures and Tables

**Table 1:** Comparison results of example 1 under different control laws.

Nominal Control	Pure Control	Dual Control of e.g., 1
369.112	491.224	289.377

**Table 2:** Comparison results of example 2 under different control laws.

Nominal Control	Pure Control	Dual Control of e.g., 2
1805.325	2156.771	1765.391

## Data Availability

Not applicable.

## References

[1] Maciej N. (2008). Optimal and suboptimal smoothing algorithms for identification of time-varying systems with randomly drifting parameters. Automatica.

[2] Nakamura S. MEMS inertial sensor toward higher accuracy & multi-axis sensing. Proceedings of the IEEE Conference on Sensors.

[3] Li J., Liu J., Zhang W.D. (2006). MEMS based micro inertial measurement system. WSEAS Trans. Circuits Syst..

[4] Wen Q., Li S. (2019). Enhanced parameterizable uncertainty to dual adaptive model predictive control. Control Theory Appl..

[5] Arcari E., Hewing L., Schlichting M., Zeilinger M. (2020). Dual stochastic MPC for systems with parametric and structural uncertainty. Learn. Dyn. Control PMLR.

[6] Ma X., Qian F., Zhang S., Wu L. (2021). Adaptive quantile control for stochastic systems. ISA Trans..

[7] Ma L., Wang Z., Hu J., Han Q. (2021). Probability-guaranteed envelope-constrained filtering for nonlinear systems subject to measurement outliers. IEEE Trans. Autom. Control.

[8] Ma X., Qian F., Zhang S., Wu L., Liu L. (2022). Adaptive dual control with online outlier detection for uncertain systems. ISA Trans..

[9] Jose A.R., Rodolfo S. (2000). Stabilization of a class of linear time-varying systems via modeling error compensation. IEEE Trans. Autom. Control.

[10] Good R., Qin S.J. Stability analysis of double EWMA run-to-run control with metrology delay. Proceedings of the American Control Conference.

[11] Yang H., Gao S., Qian F., Huang J. (2019). A Suboptimal Dual Control Method for the Stochastic Systems with Parameters Drifting. Asian J. Control.

[12] Wang L.-Y., Zhao W.-X. (2013). System identification: New models, challenges and opportunities. J. Autom..

[13] Ge S.S., Keng P.T. (2007). Approximation-based control of nonlinear MIMO time-delay systems. Automatica.

[14] Xue W., Shaojie Z., Weifang S. Optimal adaptive tracking control for a class of MIMO uncertain nonlinear systems with actuator failures. Proceedings of the 36th Chinese Control Conference.

[15] Ge S.S., Hang C.C., Zhang T. (2000). Stable adaptive control for nonlinear multivariable systems with triangular control structure. IEEE Trans. Autom. Control.

[16] Altan A., Hacıoğlu R. (2020). Model predictive control of three-axis gimbal system mounted on UAV for real-time target tracking under external disturbances. Mech. Syst. Signal Process..

[17] Karafyllis I., Krstic M. (2018). Adaptive certainty equivalence control with regulation triggered finite-time least squares identification. IEEE Trans. Autom. Control.

[18] Li C., Ding J., Lewis F.L., Chai T. (2021). A novel adaptive dynamic programming based on tracking error for nonlinear discrete-time systems. Automatica.

[19] Tse E., Bar-Shalom Y., Meier L. (1973). Wide-sense adaptive dual control for nonlinear stochastic systems. IEEE Trans. Autom. Control.

[20] Feldbaum A. (1960). Dual Control Theory, Parts I and II. Autom. Remote Control.

[21] Filatovb N.M., Unbehauen H. (2004). Adaptive Dual Control Theory and Applications.

[22] Qian F., Zhang X., Liu L., Xie G. (2020). Dual Control for Stochastic Linear MIMO Systems with Parameter Uncertainty. IEEE Access.

[23] Huang J., Qian F., Xie G., Yang H. (2016). Robust learning control for dynamic systems with mixed uncertainties. J. Syst. Eng. Electron..

[24] Milito R., Padilla C.S., Padilla R.A., Cadorin D. (1982). An innovation approach to dual control. IEEE Trans. Autom. Control.

[25] Qian F., Gao J., Li D. (2012). Complete statistical characterization of discretetime LQG and cumulant control. IEEE Trans. Autom. Control.

[26] Li D., Qian F., Fu P. (2008). Optimal nominal dual control for discrete-time LQG problem with unknown parameters. Automatica.

[27] Wang L., Qian F., Liu J. (2015). The PDF shape control of the state variable for a class of stochastic systems. Int. J. Syst. Sci..

